# A Large Deletion With a Large Impact: Homozygous 5,600 bp Deletion of the *GALNT3* Gene Causing Hyperphosphatemic Tumoral Calcinosis

**DOI:** 10.1016/j.xkme.2026.101241

**Published:** 2026-01-06

**Authors:** Julia Maria Portmann, Katharina Martini, Angela Bahr, Alexander Ritter, Carsten A. Wagner, Harald Seeger

**Affiliations:** 1 Institute of Nephrology, Zurich City Hospital, Zurich, Switzerland; 2 Department of Radiology, Hirslanden Clinic Zurich, Octorad AG, Zurich, Switzerland; 3 Institute of Medical Genetics, University of Zurich, Schlieren, Switzerland; 4 Clinic for Nephrology and Transplantation Medicine, HOCH Health Ostschweiz, Cantonal Hospital St. Gallen, St. Gallen, Switzerland; 5 Institute of Physiology, University of Zurich, Zurich, Switzerland; 6 Zurich Kidney Center, University of Zurich, Zurich, Switzerland; 7 Institute for Nephrology and Dialysis, Cantonal Hospital Baden, Baden, Switzerland; 8 Department of Nephrology, University Hospital Zurich, Zurich, Switzerland

**Keywords:** chronic kidney disease, FGF-23, hyperphosphatemia, phosphate metabolism, tissue calcification

## Abstract

Hyperphosphatemic familial tumoral calcinosis (HTC) is a rare disease caused by autosomal recessive loss of function variants in the genes encoding fibroblast growth factor 23 (FGF-23), Klotho, or GalNAc-T3. This results in reduced phosphate excretion in the renal proximal tubule, leading to hyperphosphatemia. The clinical manifestations of HTC are mainly periarticular calcifications accompanied by pain and disability, inflammation, and dental problems. Inactive forms or reduced levels of FGF-23 or resistance to the FGF-23/Klotho complex are the main pathophysiologic characteristics underlying this disease. Treatment options to reduce blood phosphate levels have only been studied in case reports and small cohorts, with positive effects from phosphate binders, acetazolamide, anti-inflammatory drugs, probenecid, nicotinamide, and sodium thiosulfate. In this report, we present the case of a 50-year-old woman with a large (at least 5,600 base pair) deletion in the gene encoding for GalNAc-T3 (*GALNT3*) who experienced bone pain during childhood and calcifications of her lower limbs at least since her mid-thirties. Intragenic *GALNT3* copy number variants have, to our knowledge, not yet been described as a cause of HTC.

## Introduction

Hyperphosphatemia is common in patients with acute or chronic kidney disease and is often accompanied by changes in calcium, fibroblast growth factor 23 (FGF-23), and parathyroid hormone levels. A very rare cause for hyperphosphatemia in the presence of normal values for parathyroid hormone, calcium, and calcitriol is hyperphosphatemic familial tumoral calcinosis (HTC), which is an autosomal recessive inherited disease leading to increased reabsorption of phosphate in the renal proximal tubule, resulting in hyperphosphatemia and ectopic calcifications. Loss of function variants in the genes encoding for FGF-23, Klotho and GalNAc-T3 cause this rare disease. FGF-23 stimulates urinary phosphate excretion by downregulating the renal phosphate transporters NaPi-IIa and NaPi-IIc.^1^ The half-life of FGF-23 is prolonged by *O*-glycosylation mediated by the enzyme polypeptide GalNAc-T3. Thus, loss of function of FGF-23 or GalNAc-T3 leads to reduced renal clearance of phosphate.^2^^,^^3^ Typically, loss of function mutations in *GALNT3* are caused by sequence variants that only affect a single or a few nucleotides.^4^ Here, we present the unique case of a 50-year-old woman with HTC with FGF-23 levels just above the lower limit of detection and a large, at least 5,600 bp deletion in the *GALNT3* gene. This is the first description of a pathogenic *GALNT3* copy number change in a patient with HTC.

## Case Report

**Figure 1:**
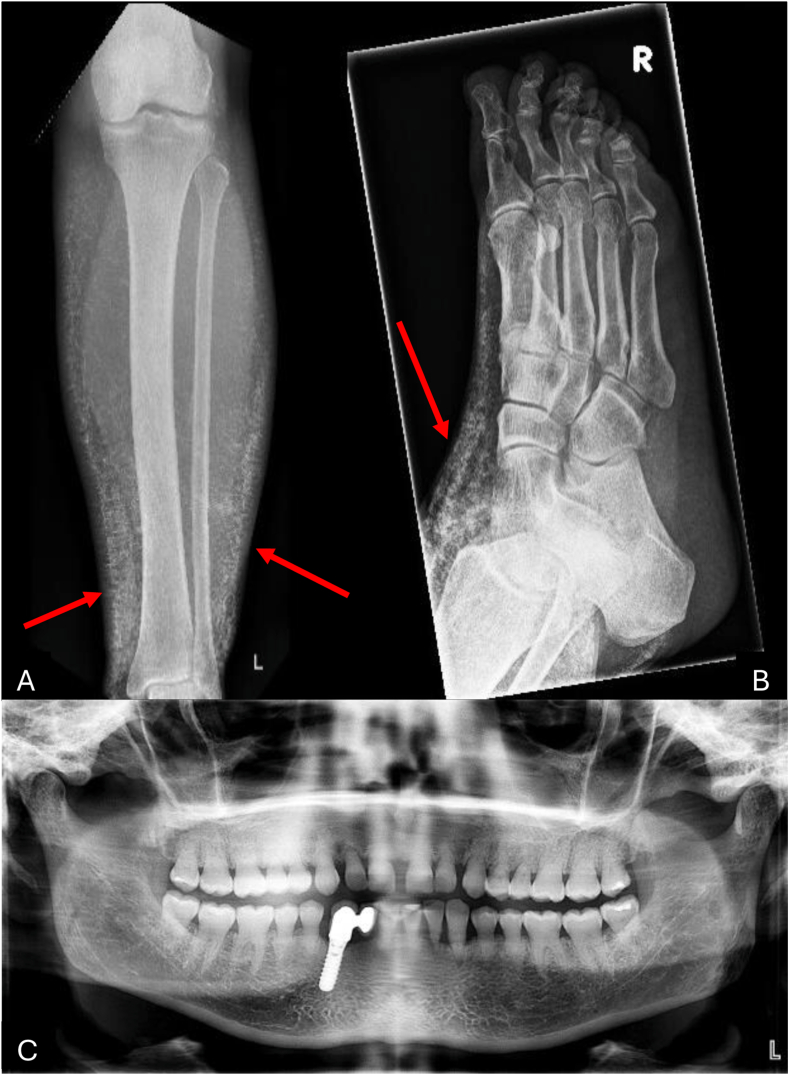
(A) X-ray of the left calf and (B) the right foot showing diffuse cutaneous and subcutaneous calcifications (red arrows). (C) Panoramic dental X-ray depicting teeth with short and bulbous roots and pulp chamber and canal obliterations.

A 50-year-old woman was referred to our nephrology outpatient clinic because of hyperphosphatemia and a slightly reduced kidney function. Blood chemistry showed serum phosphate levels of 1.85 mmol/L (normal, 0.87-1.45 mmol/L) and creatinine concentration of 85 μmol/L (estimated glomerular filtration rate by CKD-EPI [Chronic Kidney Disease Epidemiology Collaboration] equation was 70 mL/min/1.73 m^2^). She had normal values of parathyroid hormone, 1.25-dihydroxyvitamin D_3_, 25-hydroxyvitamin D_3_, and ionized calcium (Table 1). Her tubular reabsorption of phosphate was inadequately high at 93%, and her tubular maximum for phosphate normalized to glomerular filtration rate was increased at 1.73 mmol/L. The elevated phosphate level was first detected a few weeks earlier during a rheumatology consultation for joint pain in the proximal interphalangeal joint of digit V. Joint puncture displayed apatite crystals. She also mentioned that in the past 10-15 years, she had been experiencing induration of the skin, especially of her feet and lower legs. As a child, she also experienced pain in both lower legs and was diagnosed with osteomyelitis. Furthermore, she reported that she required multiple fillings and even a dental implant during early adulthood (Fig 1). Otherwise, her medical history was unremarkable, except for arterial hypertension treated with candesartan. She was on a normal diet, and her family history was negative for kidney or rheumatologic disease. There was no consanguinity.

**Table 1:** Laboratory Results

	Result	Reference Range
Creatinine, μmol/L	95	44-80
Phosphate, mmol/L	1.85	0.87-1.45
Total calcium, mmol/L	2.28	2.15-2.50
Ionized calcium, mmol/L	1.2	1.15-1.29
iPTH, ng/L	20.4	15-65
25-Hydroxyvitamin D_3_, μg/L	26.4	>20
1.25-Dihydroxyvitamin D_3,_ ng/L	70.8	19.9-79.9
Urinary phosphate, mmol/L	14.8	12.9-43.9
Urinary phosphate/creatinine ratio, mmol/mmol	1.8	0.4-4.0

Abbreviation: iPTH, intact parathyroid hormone.

Vital signs were normal except for a slightly elevated blood pressure. Physical examination revealed a painless induration of the skin on both lower legs extending to the mid calves, most prominently on the lateral aspects. Ultrasound of the kidneys was unremarkable. X-rays (Fig 1) revealed diffuse calcification of the subcutis. Doppler ultrasound showed moderate arteriosclerosis of the extracranial arteries and mediacalcinosis of the arteries in both lower legs.

Plasma intact FGF-23 was 1.24 pg/mL, just above the lower limit of detection. Intact FGF23 was measured with the human intact FGF23 enzyme-linked immunosorbent assay (#60-6600, Lot 173373, Quidel Corporation) according to the manufacturer’s protocol.^5^ Exome sequencing analysis of the genes *FGF23*, *KL*, and *GALNT3* revealed a homozygous copy number loss of at least 5,600 bp in the 2q24.3 region, encompassing exons 3, 4, and part of 5 in the glycosyltransferase domain of the *GALNT3* gene (hg19, chr2:166,615,961-166,621,566). A deletion of these coding regions results in a premature stop codon, likely leading to a non-functional allele product (hg19 NM_004482.4:c.516_958del p.(Glu172Aspfs∗6)).

The patient was treated with a phosphate-reduced diet, and therapy with sevelamer hydrochloride was initiated. During the 3-year follow-up, kidney function remained stable and phosphate levels varied between 1.6-2.0 mmol/L. Phosphate reduction into the normal range was not accomplished. No clinical changes regarding soft tissue and vascular calcifications were noted on follow-up X-ray.

## Discussion

**Figure 2:**
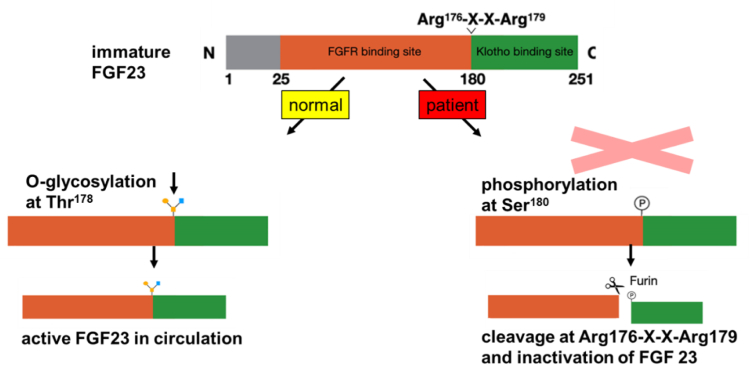
Impact of GalNAc-T3 enzyme dysfunction; normal situation (left), deletion leading to GalNAc-T3 dysfunction (right). FGF-23, fibroblast growth factor 23; FGFR, fibroblast growth factor receptor.

HTC, an autosomal recessive inherited disease, is a rare cause of hyperphosphatemia in patients with normal estimated glomerular filtration rate, leading to ectopic calcifications.^6^ FGF-23, a 251-amino acid peptide^7^ that is mainly produced by osteocytes and osteoblasts^8^ is one of the main players in phosphate homeostasis. The active form of FGF-23 binds to the FGF-1 receptor and its coreceptor α-Klotho in the proximal tubule. This leads to phosphaturia via downregulation of the sodium/phosphate cotransporters NaPi-IIa und NaPi-IIc.^1^^,^^7^^,^^8^ The 1-α-hydroxylase activity is inhibited by FGF-23, resulting in lower levels of 1.25-dihydroxyvitamin D_3_.^8^ The enzyme GalNAc-T3 catalyzes posttranslational *O*-glycosylation of FGF-23.^9^ The absence of this process leads to increased cleavage of FGF-23 into inactive fragments (Fig 2).^7^ In HTC, mutations in the genes encoding FGF-23, GalNacT3, and α-Klotho result in a reduced level of FGF-23 (*FGF-23* or *GALNT3* loss of function variants), an inefficient form of FGF-23 (*FGF-23* loss of function variants), or FGF-23 resistance at the FGF-23-FGFR1c-Klotho complex (*KL* loss of function variants). Hyperphosphatemia is caused by enhanced phosphate reabsorption in the proximal tubule and by increased gastrointestinal phosphate absorption because of inappropriately normal or elevated 1.25-dihydroxyvitamin D_3_ levels. This leads to the clinical phenotype of HTC.^8^^,^^10^, ^11^, ^12^ Our patient had a normal 1.25-dihydroxvitamin D_3_ level (Table 1) despite low FGF-23. A possible explanation could be that the inhibitory effect of hyperphosphatemia on 1-α-hydroxylase activity^13^ may, in some patients with HTC, outweigh the stimulatory effect expected from low FGF-23 levels.^2^ In our patient, we only measured intact FGF-23, which is the biologically active form and relevant for maintaining phosphate homeostasis. Levels of the C-terminal FGF-23 fragment were likely elevated, but unfortunately, C-terminal FGF-23 could not be assessed, which is a limitation of this report.

Our patient had moderate clinical symptoms with calcifications of both lower limbs. In addition, arterial calcifications were noted in both thighs. She also had dental problems with the need for a dental implant and experienced joint pain in proximal interphalangeal joint 5 due to intra-articular apatite crystals. She had chronically increased C-reactive protein levels in the range of 6-9 mg/L.

Typical manifestations of HTC are calcifications in the skin or subcutaneous tissue, mainly in periarticular locations (especially the lateral hips). Calcifications have also been found in bones, joints, and even the submucosal lining of the large intestine.^7^^,^^11^ Calcifications are usually present in areas with tissue hypoxia, inflammation, or repetitive trauma^7^ and consist of hydroxyapatite and/or calcium carbonate.^7^^,^^14^ The involvement and severity of calcifications differ significantly between patients. A unique clinical feature of HTC is hyperostosis; patients experience pain in areas overlying the diaphyseal regions of long bones. This is often—as in our patient—misdiagnosed as osteomyelitis. Some patients have clinical signs of systemic inflammation; in these groups, elevated C-reactive protein levels are not uncommon. Patients with HTC exhibit calcification of both large and small vessels, such as cardiac calcification and peripheral vascular disease,^7^ which can result in severe peripheral ischemia, potentially leading to amputation.^15^ Dental involvement is the most common HTC-defining manifestation, as it can be observed even without other ectopic calcifications. Typical dental abnormalities include pulp chamber and root canal obliterations, pulp stones, short and bulbous roots, enamel hypoplasia and root dilacerations.^6^^,^^16^ Just recently, it was shown that chronic inflammation associated with HTC can result in amino acid amyloidosis.^17^

In patients with typical clinical symptoms and conspicuous blood results (a combination of hyperphosphatemia, high-normal calcium, low-normal parathyroid hormone, inappropriately normal or elevated 1.25-dihydroxyvitamin D_3_), a diagnosis of HTC must be considered. The gold standard to diagnose HCT is genetic testing.^7^ The key clue in our patient prompting further investigation into HTC was hyperphosphatemia. However, it ultimately took several years of enduring health issues before the proper diagnosis was made.

*GALNT3* is located on 2q24.3, and the link between HTC and genetic variants in this region was discovered in 2004.^10^ In the last few years, several new disease-causing mutations have been reported.^4^^,^^18^, ^19^, ^20^, ^21^, ^22^ These mainly comprise single nucleotide changes in the coding sequence, both missense and truncating variants, leading to loss-of-function of the GalNac-T3 enzyme.^6^ In our case, a homozygous copy number loss of at least 5,600 bp, leading to an out-of-frame deletion of exons 3, 4, and part of 5 of the *GALNT3* gene, was detected. This is the largest deletion and the first reported copy number change in the *GALNT3* gene associated with HTC to date. Intragenic deletions arise through several mechanisms affecting DNA replication, repair, or recombination. Recurrent deletions often involve extended homologous sequences, such as low copy repeats, which mediate nonallelic homologous recombination. Nonrecurrent deletions, as in the *GALNT* case, display variable breakpoints with minimal or no homology and often occur near low copy repeat-rich regions, predisposing to local instability.^23^ Concerning the *GALNT3* deletion, sequences of repetitive mobile elements are present in introns 2 and 4, which may have facilitated aberrant recombination or replication events. So far, no genotype-phenotype correlation has been found for truncating variants in *GALNT3*,^19^ and even in family members with the same genotype, the clinical manifestations differ significantly.^11^^,^^19^

At present, controlled trials for treatment options are lacking due to the rarity of the disease. In the absence of a curative treatment, the therapeutic goal is to reduce plasma phosphate and alleviate symptoms. In case reports or series in the literature, several patients responded to reduced dietary phosphate intake, treatment with non–calcium-containing phosphate binders, probenecid, acetazolamide, and nicotinamide.^6^^,^^7^^,^^11^ Therapy with probenecid and acetazolamide led to an increased renal phosphate excretion. The mechanism underlying probenecid’s activity is unknown, but acetazolamide causes a proximal renal tubular acidosis, resulting in enhanced phosphate excretion.^11^ In a case study by Ramnitz et al^11^ reporting a small number of patients with HTC and elevated C-reactive protein, therapy with anakinra (IL-1 receptor antagonist) and/or canakinumab (IL-1β antibody) had positive effects on inflammation, well-being, energy level, and appetite.^11^ In one case report, the use of teriparatide led to increased renal phosphate excretion and a decrease in phosphatemia,^24^ and topical sodium thiosulfate was reported to be successful in treating cutaneous calcifications.^25^ No differences in treatment response have been observed across different genotypes.^6^

## Conclusions

HTC is a very rare cause of hyperphosphatemia that must be considered after excluding more common causes. Early diagnosis is essential to prevent calcifications of blood vessels, preserve joint mobility, reduce pain, and avoid dental procedures.
